# Towards an Enhanced Tool for Quantifying the Degree of LV Hyper-Trabeculation

**DOI:** 10.3390/jcm10030503

**Published:** 2021-02-01

**Authors:** Gregorio Bernabé, José D. Casanova, Josefa González-Carrillo, Juan R. Gimeno-Blanes

**Affiliations:** 1 Computer Engineering, University of Murcia, 30100 Murcia, Spain; josedavid.casanova@um.es; 2 Unidad CSUR de Cardiopatías Familiares, Servicio de Cardiología, Hospital Virgen of Arrixaca, El Palmar, 30120 Murcia, Spain; josegonca.alarcon@gmail.com (J.G.-C.); jgimeno@secardiologia.es (J.R.G.-B.)

**Keywords:** genetic cardiomyopathies, trabeculae, left ventricle, automatic quantification, parallelization, comparison, accuracy

## Abstract

Left ventricular non-compaction (LVNC) is defined by an increase of trabeculations in left ventricular (LV) endomyocardium. Although LVNC can be in isolation, an increase in hypertrabeculation often accompanies genetic cardiomyopathies. Current methods for quantification of LV trabeculae have limitations. Several improvements are proposed and implemented to enhance a software tool to quantify the trabeculae degree in the LV myocardium in an accurate and automatic way for a population of patients with genetic cardiomyopathies (QLVTHCI). The software tool is developed and evaluated for a population of 59 patients (470 end-diastole cardiac magnetic resonance images). This tool produces volumes of the compact sector and the trabecular area, the proportion between these volumes, and the left ventricular and trabeculated masses. Substantial enhancements are obtained over the manual process performed by cardiologists, so saving important diagnosis time. The parallelization of the detection of the external layer is proposed to ensure real-time processing of a patient, obtaining speed-ups from 7.5 to 1500 with regard to QLVTHCI and the manual process used traditionally by cardiologists. Comparing the method proposed with the fractal proposal to differentiate LVNC and non-LVNC patients among 27 subjects with previously diagnosed cardiomyopathies, QLVTHCI presents a full diagnostic accuracy, while the fractal criteria achieve 78%. Moreover, QLTVHCI can be installed and integrated in hospitals on request, whereas the high cost of the license of the fractal method per year of this tool has prevented reproducibility by other medical centers.

## 1. Introduction

The American Heart Association considers LVNC as a distinct primary genetic cardiomyopathy caused by an abnormal compaction process of the developing myocardium [1]. In contrast, LVNC is defined as an unclassified cardiomyopathy by the European Society of Cardiology [2] because LVNC could appear as a morphological trait of other cardiomyopathies, especially hypertrophic cardiomyopathy (HCM). Whether LVNC is a distinct cardiomyopathy or a morphologic trait shared by different types of cardiomyopathies is still debated [3,4,5]. Cardiomyopathies in general, and LVNC in particular, are thought to be caused by genetic mutations [6]. Cardiomyopathies are a cause of significant morbidity and mortality in affected individuals at all ages [7]. Of note, these conditions are the main cause of sudden cardiac death in the young and athletes. Moreover, LVNC is an early marker of disease in preclinical/silent mutation carriers from families with hypertrophic cardiomyopathy, dilated cardiomyopathy (DCM), and arrhythmogenic cardiomyopathy (ARVC). Systolic impairment and hypertrabeculation have been associated with an increased risk of thromboembolic events in DCM [1,8]. Left ventricular crypts, clefts, and other forms of hypertrabeculation can be seen in up to 70-80% of HCM mutation carriers [9,10,11].

There is controversy about the accuracy of different methods for quantifying the non-compact myocardium [12]. To date, there are four echocardiographic and two cardiac magnetic resonance (CMR) imaging-based approaches, which differ in the diagnostic ratio of compact to non-compact myocardium and the timing of measurement (end-systole or end-diastole) [5].

Although there is some agreement on the preferred diagnostic criteria of LVNC based on echocardiographic [13] or CMR images [14,15], based on the measurement of the ratio between the thickness of the non-compact to compact layer of the myocardium in different segments in end-diastole, the estimation of the non-compact myocardium by endocardium delineation from CMR images has been recently proposed to quantify the non-compact mass. Recently, a method based on fractals [16,17] introduced the fractal dimension (FD) to measure the tortuosity of endocardial borders. Therefore, this tool introduces complexity, avoiding its usability by other medical centers or hospitals.

**Figure 1:**
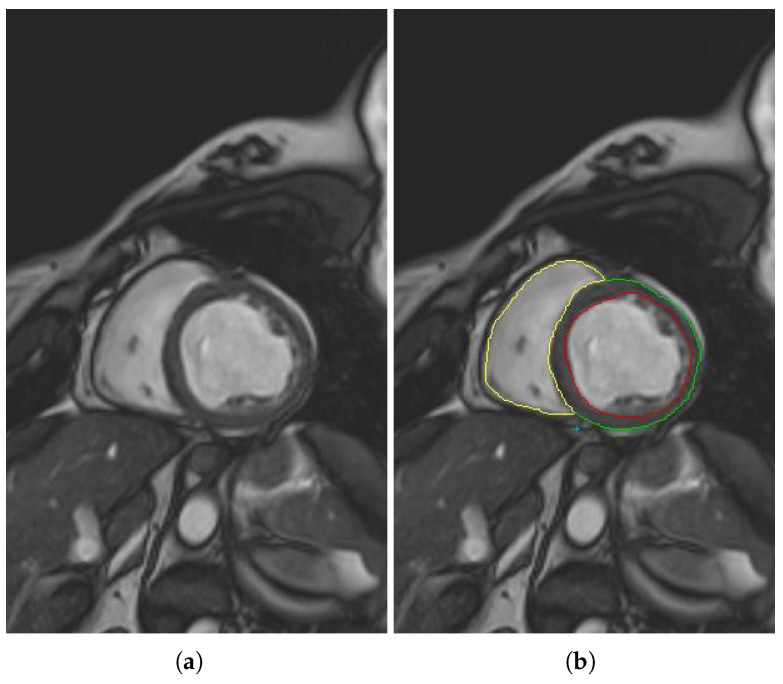
(**a**) Input cardiac magnetic resonance (CMR) slice. (**b**) Trabeculae included in the LV cavity (black delimited zone), RV cavity (yellow delimited zone), and external layer of the compact zone (yellow and green line).

Traditionally, radiologists and cardiologists manually identify on a cardiac magnetic resonance of a patient, as shown in Figure 1a, the LV cavity where the trabeculae are included (red delimited zone in Figure 1b), the right ventricle of the myocardium (yellow delimited zone in Figure 1b), and the external layer of the myocardium (green and yellow delimited zone in Figure 1b). The trabecular zone is characterized by several black areas inside the LV, whereas the zone located between the external layer of the myocardium and the LV cavity is called the compact zone. Once the different structures have been detected, the quantification of the trabeculae is obtained as the relation of the trabecular area with respect to the compact zone as in previous studies [18,19].

In [20,21,22], we proposed, evaluated, and updated a software tool to quantify the degree of hyper-trabeculation automatically in the LV myocardium including patients with dilated and hypertrophic cardiomyopathies (QLVTHC). Based on artificial vision techniques, the proposal identifies LV and RV cavities, the external layer of the myocardium, and the trabecular area to determine the percentage quantification of the trabecular area with regard to the compact zone.

Our main goal is to obtain the percentage quantification of trabeculation in the LV myocardium in a series of patients with genetic cardiomyopathies like non-compacted cardiomyopathy, RV or LV arrhythmogenic cardiomyopathy, DCM, HCM, unclassifiable or mixed cardiomyopathies, and inherited cardiomyopathies. Therefore, our automatic tool [20] is improved to process patients with genetic cardiomyopathies (QLVTHCI). The input of QLVTHCI is the different slices obtained for a particular patient based on end-diastolic CMR images. Manual tuning by cardiologists of different parameters is required to identify the LV cavity, detect the trabecular zones in the interior of the LV cavity, and accurately reach and establish the external layer. QLVTHCI processes each slice and obtains the left ventricle trabecular myocardial mass (TM) and compact mass (compact myocardium (CM)) to produce the percentage of trabeculated myocardium (TM%). Some improvements with regard to our previous tool (QLVTHC) are proposed, such as the detection of different maximally stable extremal regions (MSERs) in a centered ROI to automatically determine the LV cavity, the accurate and reliable detection of the RV cavity at any location, the refining of the search to obtain the external layer of the myocardium and the trabecular areas, and the processing of different slices in a reverse order allowing the proposed method to process patients with different cardiomyopathies efficiently. The enhanced method achieves considerable time savings compared to the manual process, so minimizing the possible human error. A parallelization of the detection of the external layer of the myocardium is proposed to process each slice of a patient in real time, improving speed-ups significantly. Moreover, other different patients with LVNC cardiomyopathy will be processed by QLVTHCI and fractal analysis [16,17] to determine better diagnostic accuracy.

The remainder of this paper is divided into four sections. The improvements of the software tool developed (QLVTHCI) , the parallelization of the detection of the external layer, a brief description of fractal analysis, the populations, the magnetic resonance protocol, and the procedure to determine the quality of medical images by radiologists and cardiologists are presented in Section 2. To test the enhanced tool, we show in Section 3 several patients with genetic cardiomyopathies, where different and representative outputs are shown. Moreover, the results of the parallelization in the QLVTHCI method are presented. A comparison of several patients with QLVTHCI and the method proposed by Captur [16,17] are performed in Section 4. Finally, Section 5 outlines the paper, indicates the main conclusions, and advances future work.

## 2. Methods

In this section, the improvements of the enhanced method developed (QLVTHCI) are presented, the parallelization of the detection of the external layer is developed, a brief description of the fractal analysis is introduced, the description of the populations used in this work is detailed, the magnetic resonance protocol is described, and the procedure to determine the quality of medical images by cardiologists is introduced.

### 2.1. A Software Tool to Quantify the Trabeculae Degree in the LV Myocardium for a Population of Patients with Genetic Cardiomyopathies (QLVTHCI)

Other genetic cardiomyopathies like spongiform, RV or LV arrhythmogenic, Brugada syndrome, sudden arrhythmic death syndrome, unclassifiable, or mixed cardiomyopathies could be revealed in many patients. Therefore, the different heart parts of the myocardium can present irregular features or different structures to DCM or HCM. Thus, different improvements are proposed for the method [20] to enhance the software tool for the quantification of the trabeculae degree in the LV myocardium in an accurate and automatic way in mixed cardiomyopathy patients (QLVTHCI):

- The DICOM format is integrated to read the input images, which are the different slices obtained for a particular patient based on end-diastolic CMR images. The thickness of each slice (in mm), the spacing between slices (in mm), and the pixel spacing are automatically determined.
- The different MSERs are detected in a centered ROI of each input image by the use of OpenCV [23]. As the LV cavity is normally represented by a circular shape, the centroid of each MSER detected is computed in order to automatically identify the left ventricle cavity anywhere in the image and for applying the convex hull.
- The previous application of the convex hull allows a second refining to optimize the search process of the external layer and the trabeculae areas. The parameter e-expand is redefined and adjusted to accurately determine the external layer of the compact zone, thanks to plotting several lines from the centroid of the LV to reach the points of the external layer. This parameter establishes the distance of the lines between the centroid of the LV cavity and the possible space where the external layer can be found, taking into account the particular features of genetic cardiomyopathies. We optimized the parameter e-expand for different situations or possible cardiomyopathies.
- The accurate and reliable detection of the RV cavity at any location of a slice.
- The automatic processing of the slices stored in reverse order (from basal to apical).

These enhancements complete the tool to process different inherited cardiomyopathies efficiently.

Therefore, for each slice processed, QLVTHCI detects the LV cavity, the RV cavity, the external layer of the myocardium, and the trabecular zones in the interior of the LV cavity. QLVTHCI obtains the total areas occupied by the LV cavity, the trabecular contours in the interior of the LV cavity, and the compact zone. The percentage quantification of the trabecular zone is the area of the trabecular zone divided by the sum of the areas of the compact zone and the trabecular zone. QLVTHCI finally computes the total volumes of the trabecular zone and the compact zone for a particular patient. The percentage quantification of the trabecular zone is the volume of the trabecular zone divided by the sum of the volumes of the compact zone and the trabecular zone. The cutoff for the percentage quantification of trabeculated myocardium was 27.4% [22] to differentiate LVNC and non-LVNC patients. The mass (in grams) of the compact zone and the trabecular zone was obtained using the established density.

In a quick and automatic way, cardiologists obtain the different structures identified by the software tool and the degree of trabeculation of the LV accurately for a determined patient with dilated, hypertrophic, and some inherited cardiomyopathies or mixed cardiomyopathies (QLVTHCI).

### 2.2. Parallelization Of QLVTHCI

In [20], we theoretically and experimentally analyzed the execution time of the self-optimized software tool of QLVTHC. Similarly, the detection of the external layer is the most expensive phase of QLVTHCI. This step consists of a loop where different lines are drawn between the centroid of the LV cavity and the possible space where the points of the external layer are found. Therefore, this phase depends on the length of the compact zone perimeter, 2∗π∗r, where *r* is the ratio of the LV cavity (*r* is about *n*/3000). If *l* is the length of each line plotted from the center of the LV to reach the external layer (l=r∗d, where *d* is about 1.60), then the cost of this phase is O(2∗π∗r∗l), that is $O(n^{2})$. Then, this phase has a major contribution to the execution time (around 50% of the total execution time) of the QLVTHCI method. Therefore, we parallelized the detection of the external layer of the compact zone with OpenMP to process each slice of the input in less than 1 second.

### 2.3. The Fractal Analysis

In the method developed by Captur et al. [16,17], fractal analysis, was performed on the end-diastolic frames of each short-axis slice in the LV stack. The analysis was divided into two parts: image segmentation to extract the endocardial border and then the calculation of the FD of the endocardial border using the box-counting method.

To assess the global LV FD, the FDs from each slice in the LV were averaged. To assess local fractal characteristics, the maximal FD in the basal, mid, and apical thirds of the LV were recorded. The optimum diagnostic threshold for global FD LV was 1.26 [16] to differentiate LVNC and non-LVNC patients, whereas the maximal FD was 1.30 [16] to differentiate the LVNC and non-LVNC thirds of slices.

### 2.4. Populations

Each patient had different cardiac images or slices obtained by magnetic resonance. Two set of patients were used in this paper:

- A set of 59 patients (identified from X1 to X59) with previously diagnosed cardiomyopathies such as non-compacted cardiomyopathy, RV or LV arrhythmogenic cardiomyopathy, DCM, HCM, unclassifiable or mixed cardiomyopathies, and inherited cardiomyopathies.
- A group of 27 patients (identified as P1 to P27) with previously diagnosed LVNC cardiomyopathy meeting Petersen’s criteria [14].

All participants were recruited from an inherited cardiomyopathy clinic. Patients with an available good quality CMR study were included. The first CMR test was used for the study.

### 2.5. Magnetic Resonance Protocol

The different images or slices of each patient were acquired by CMR in the Hospital Mesa de Castillo (HMC) and University Hospital Virgen de la Arrixaca (UHVA), both in Murcia (Spain). The main characteristics of the scanners are shown in Table 1. In both scanners, images were obtained in synchronization with the ECG and in apnea. Moreover, the LV function was assessed with a free precision in standard stable balance.

### 2.6. Quality Evaluation by Medical Experts

Cardiologists evaluated the image outputs of the QLVTHCI with the different structures of the heart detected in order to determine the quality of medical images. These experts scored the slices, using the scale from 1.0 to 5.0 in intervals of 0.5, proposed by Gibson et al. [24], where the upper value identifies an exact match (without differences) and the lower value determines large diagnostically significant differences. Scores ≥ 3.5 mark the border between non-significant diagnostic losses (4.0 = differences are not noticeable) and significant (3.0 = small and 2.0 = medium).

The different cardiac magnetic resonance images or slices of each patient and the image outputs of the QLVTHCI were visualized and compared by cardiologists.

## 3. Results

We applied the enhanced proposal described in the previous section to a set of 59 patients (identified from X1 to X59) with previously diagnosed cardiomyopathies like non-compacted cardiomyopathy, RV or LV arrhythmogenic cardiomyopathy, DCM, HCM, unclassifiable or mixed cardiomyopathies, and inherited cardiomyopathies.

Mean CM was 102.3 ± 33.1 g and TM 47.0 ± 15.3 g. Mean TM% from the total was 31.8% ± 5.9%, using the software proposed in the previous section (QLVTHCI) for the automatic delineation of borders.

**Figure 2:**
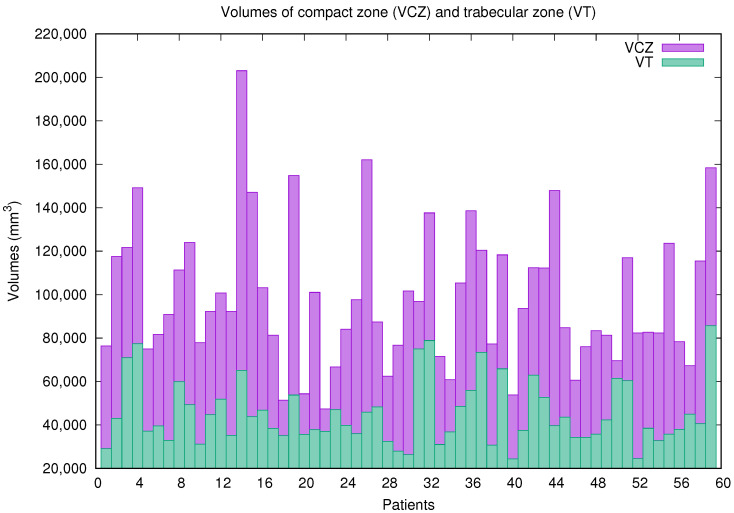
Volume of the compact zone (VCZ) and volume of the trabecular area (VT) computed by QLVTHCI for patients (X1–X59).

**Figure 3:**
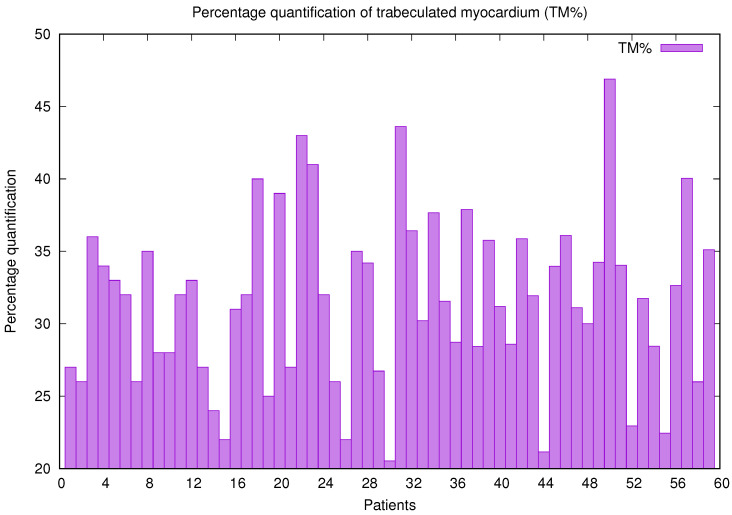
Percentage quantification of trabeculated myocardium (TM%) computed by QLVTHCI for patients (X1–X59).

In Figure 2, the volume of the compact zone (VCZ) and the volume of the trabecular area (VT) for each patient (470 slices or cardiac images) with previously diagnosed inherited cardiomyopathies are shown. Both volumes cover a wide range of values or sizes, demonstrating that the enhanced method adjusts and adapts to different situations efficiently. Through the computation of both volumes, QLVTHCI easily calculates the percentage quantification of trabeculated myocardium (TM%), presented in Figure 3. Finally, the left ventricular and trabeculated masses are computed automatically from a given density.

Table 2 shows the mean score of two cardiologists to obtain the different structures of the heart (LV and RV cavities, trabecular and compact areas) performed by the enhanced method. Cardiologists determined the quality of the output images to be very good in all slices due to the different improvements proposed in this paper. The software tool proposed allowed these medical experts to establish a diagnosis considering a precise percentage quantification of the trabeculations and having the masses and volumes of the trabeculae and compact areas available in a short time. All evaluated images were marked with scores ≥ 3.5. Therefore, there were no non-significant diagnostic losses, and differences were not noticeable for diagnosis. Moreover, the vast majority of the slices evaluated were identical to the original inputs (93.19%) according to the cardiologists (scores = 5.0). Furthermore, the rest of the slices tested (6.80%) were practically exact for a correct diagnosis (scores = 4.5 or = 4.0). The weighted kappa statistic showed an agreement between the two observers of 96.6% (kappa 0.76).

Therefore, QLVTHCI is ready to operate and adapt to different inherited myocardiopathies or different situations with mixed myocardiopathies. Moreover, the tool helps issue several diagnoses and analyze the different heart parts in diverse positions and the input images coming from different hospitals.

**Figure 4:**
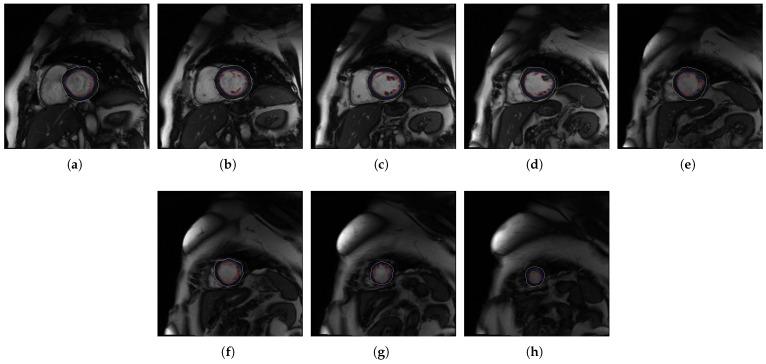
(**a**–**h**) Slices 1 to 8 of X5.

**Figure 5:**
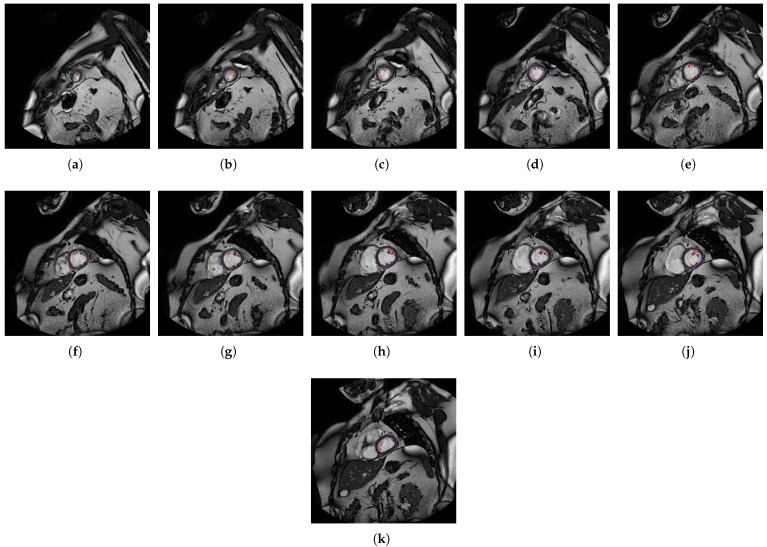
(**a**–**k**) Slices 1 to 11 of X20.

**Figure 6:**
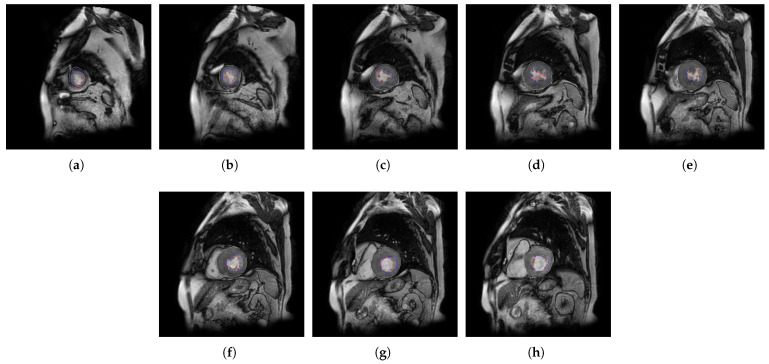
(**a**–**h**) Slices 1 to 8 of X14.

**Figure 7:**
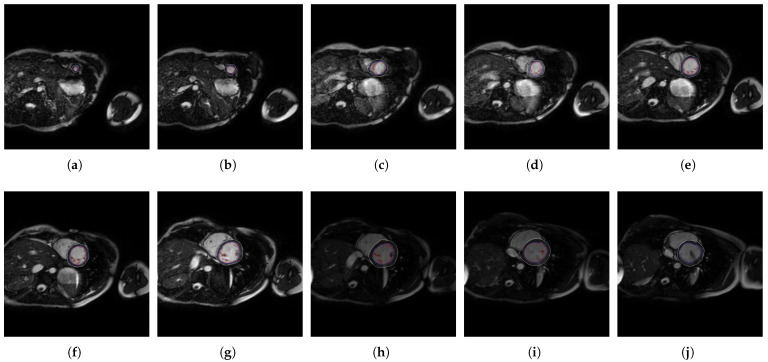
(**a**–**j**) Slices 1 to 10 of X10.

In Figure 4, Figure 5, Figure 6 and Figure 7, the output slices of QLVTHCI are presented for X5, X20, X14, and X10, respectively. In each output slice, the compact zone is delimited between the external layer (in gray) and the trabeculated zone (in blue), whereas the LV cavity without trabeculae is also delimited (in red). X5 was previously diagnosed with RV arrhythmogenic, and the slices appeared in a reverse order, whereas X20 was previously diagnosed with a mixed DCM and spongiform cardiomyopathies, and the LV was not always in the center of the slice, while the pixel values were very different with regard to X5. On the other hand, X14 and X10 were previously diagnosed with hypertrophic and Brugada syndrome cardiomyopathy, respectively. The different structures of the heart and the pixel values were very different with respect to the previous cases. All these slices of these patients were evaluated by cardiologists with a score of 5.0, so demonstrating QLVTHCI that is able to adapt and detect different forms of compact zones and trabecular zones accurately, the positions in a slice, the reverse order of the slices to compute the volumes of compact and trabecular zones, several or mixed cardiomyopathies, and very different pixel values.

The enhanced tool QLVTHCI allows medical experts to obtain in an automatic way precise measurements of the volumes and masses of the trabecular area and compact zone. Moreover, the percentage quantification of trabeculated myocardium was obtained to easily differentiate LVNC patients and non-LVNC patients. In the automatic method, the output of each slice was obtained in less than 10 s; therefore, a patient is processed in less than one or two min. However, in the traditional process, cardiologists take around 25 min per slice.

## 4. Comparison

We executed QLVTHCI with the parallelization of the detection of the external layer, shown in Section 2, and the method developed by Captur [16,17] on a group of 27 patients (identified as P1 to P27) with previously diagnosed LVNC cardiomyopathy, obtained by magnetic resonance studies performed at the two hospitals mentioned in Section 3. We executed both methods on a four core Intel^®^ CPU i7-4700MQ with hyperthreading running at 2.40 GHz.

In both proposals, the execution time required to process each slice of the input can be less than one second. Therefore, a patient can be processed in real time. Table 3 shows both the global FD obtained by the Captur method and the percentage quantification of trabeculated myocardium (TM%) obtained by QLVTHCI for 27 patients.

The optimum diagnostic threshold for global FD LV was 1.26 [16], whereas the cutoff for the percentage quantification of trabeculated myocardium (TM%) was 27.4% [22] to differentiate LVNC and non-LVNC patients. Therefore, all patients were marked as LVNC by QLVTHCI. However, of the 27 patients, six (P4, P7, P13, P15, P20, and P27) were mislabeled as healthy or normal by the Captur method. This is due to the compact myocardium not being evaluated in this method, and the global FD represents the tortuosity measured by the pixelation of the line of the endocardial borders. Table 4 shows both the FD and the TM% of these patients for each slice divided into basal (B1, B2, B3), middle (M1, M2, M3), and apical (A1, A2, A3) thirds. The Captur proposal also computed the maximal FD in the basal, mid, and apical thirds, and the optimum diagnostic threshold for the maximal FD was 1.30 [16] to differentiate the LVNC and non-LVNC thirds of slices. Now, the identification was not correct as the LVNC slice by the Captur method in the B2, M1, A1, and A3 slices of P4, in the B1, B3, M1, M2, M3, and A2 slices of P7, in the M1, M2, A1, A2, and A3 slices of P13, in the B1, B2, B3, M1, A1, A2, and A3 slices of P15, in the B1, B2, and B3 slices of P20, and in the A1, A2, A3, and B3 slices of P27. This local diagnostic marker for LVNC solved and determined as LVNC the basal and apical thirds of P4, the apical third of P7, and the mid third of P13. However, the mid third of P4, the basal and mid thirds of P7, the apical third of P13, the basal, mid, and apical thirds of P15, the basal third of P20, and the apical and basal thirds of P27 continued to be marked as non-LVNC, obtaining an accuracy lower than QLVTHCI. On the other hand, both methods labeled as non-LVNC the slices B1 of P4, B2 of P7, B1 and B2 of P13, M3 of P15, A1, A2, and A3 of P20, and A1 and A2 of P27, demonstrating that healthy slices can be differentiated for LNVC.

Moreover, the high cost of the license of the fractal method per year due to the complexity of this tool has prevented reproducibility by other medical centers or hospitals. Conversely, QLTVHCI could be used and integrated into medical centers on request.

## 5. Conclusions

An enhanced tool to quantify the trabeculae in an automatic way identifying the LV cavity, the RV cavity, and the compact and trabeculated zones from cardiac magnetic resonance slices is shown to perform accurately and adapt efficiently in a population of 59 patients with several genetic cardiomyopathies. Substantial enhancements are obtained over the manual process performed by cardiologists, so saving important diagnosis time. The different slices of patients (470 slices) obtained by magnetic resonance are the inputs, which come from different scanners and hospitals. Understandable and precise measurements of the areas, volumes, and masses of the trabecular area and compact zone, as well as the percentage quantification of trabeculated myocardium are produced in real time by adjusting different parameters. Several outputs are shown with different genetic cardiomyopathies like RV arrhythmogenic, DCM and spongiform mixed, HCM or Brugada syndrome to demonstrate the efficiency of the tool. The percentage quantification of the trabeculated myocardium varies from 20.53 to 46.90, demonstrating that QLVTHCI covers a wide range of trabecular and compact zones from inconsiderable to substantial values in patients with different or mixed genetic cardiomyopathies. The parallelization of the detection of the external layer of the compact zone is proposed to ensure the real-time analysis per slice in a patient. Therefore, important speed-ups are obtained ranging from 7.5 to 1500 with regard to QLVTHCI and the manual process used traditionally by cardiologists.

In subjects with previously diagnosed cardiomyopathies, the percentage quantification of the trabeculated myocardium measured by QLVTHCI distinguishes LVNC from healthy with higher diagnostic accuracy than the biological signal obtained by fractal analysis. Moreover, QLVTHCI presents a lower cost than the method developed by Captur.

The quantification of the trabeculae in the RV of the myocardium is an open line of research where the enhanced method proposed can be applied. The software tool is available to integrate into others medical centers or hospitals on request.

## Figures and Tables

**Table 1:** Characteristics of the scanners. HMC, Hospital Mesa de Castillo; UHVA, University Hospital Virgen de la Arrixaca.

Hospital	HMC	UHVA
Analysis	AW4.3-08	View-Forum 6.3
Scanner	1.5 T scanner	1.5 T magnet
Provider	General Electric Systems	Philips Medical Systems
Repetition interval	3.8 ms	3.3 ms
Echo time	1.7 ms	1.7 ms
Flip	60°	60°
Matrix	224 × 224	192 × 256
Echo train length	23	23
Cutting thickness	8 mm	8 mm
Space between slices	2 mm	2 mm
Phases	20	20

**Table 2:** Average quality measure obtained from medical experts for slices with different heart parts identified by QLVTHCI.

	5.0	4.5	4.0	3.5	3.0	2.5	2.0	1.5	1.0
QLVTHCI	93.19%	5.74%	1.06%	0.00%	0.00%	0.00%	0.00%	0.00%	0.00%

**Table 3:** Results of global fractal dimension (FD) [16] and percentage quantification of trabeculated myocardium (QLVTHCI).

Patient	FD	TM%	Patient	FD	TM%	Patient	FD	TM%
P1	1.37	38.51	P10	1.34	28.99	P19	1.34	31.73
P2	1.31	31.43	P11	1.26	28.62	P20	1.24	28.78
P3	1.28	32.29	P12	1.31	34.31	P21	1.31	34.06
P4	1.21	31.51	P13	1.20	37.34	P22	1.32	36.30
P5	1.33	44.72	P14	1.27	47.35	P23	1.27	29.04
P6	1.30	40.73	P15	1.21	33.98	P24	1.38	32.20
P7	1.23	37.28	P16	1.37	34.71	P25	1.30	29.14
P8	1.39	31.02	P17	1.27	30.94	P26	1.33	36.33
P9	1.28	27.40	P18	1.27	30.15	P27	1.18	32.10

**Table 4:** Results of FD [16] and TM% (QLVTHCI) for each slice of patients P4, P7, P11, P13, P15, P20, and P27.

	Basal			Middle			Apical		
Patient	B1	B2	B3	M1	M2	M3	A1	A2	A3
P4-FD	1.08	1.17	1.32	1.27			1.21	1.31	1.11
P4-TM%	19.15	31.14	36.11	30.39			40.69	29.88	29.96
P7-FD	1.10	1.14	1.20	1.20	1.13	1.19	1.31	1.27	1.44
P7-TM%	28.61	21.14	39.59	44.45	44.60	38.53	32.41	48.67	34.36
P13-FD	1.13	1.21	1.35	1.27	1.28	1.31	1.24	1.06	1.11
p13-TM%	0.81	24.77	33.29	44.75	46.19	49.17	51.89	43.59	30.38
P15-FD	1.22	1.26	1.23	1.18		1.18	1.21	1.17	1.27
P15-TM%	48.14	43.79	37.44	35.61		25.12	31.16	45.73	30.33
P20-FD	1.17	1.23	1.20	1.33		1.33	1.21	1.20	1.22
P20-TM%	35.44	36.20	31.02	41.35		34.06	25.64	18.21	18.87
P27-FD	1.19	1.17	1.17				1.18	1.25	1.14
P27-TM%	33.83	36.73	34.61				26.01	22.46	35.93

## Data Availability

The data used to support the findings of this study are restricted by the local ethical committee in order to protect patient privacy. Data are available from the corresponding author upon request for researchers who meet the criteria for access to confidential data.
